# Non-vitamin K antagonist oral anticoagulants in patients with an increased risk of bleeding

**DOI:** 10.1007/s00508-018-1381-5

**Published:** 2018-08-20

**Authors:** Thomas Gremmel, Alexander Niessner, Hans Domanovits, Martin Frossard, Gürkan Sengölge, Barbara Steinlechner, Thomas Sycha, Michael Wolzt, Ingrid Pabinger

**Affiliations:** 1 Department of Internal Medicine II, Medical University of Vienna, Währinger Gürtel 18–20, 1090 Vienna, Austria; 2 Department of Internal Medicine, Cardiology and Nephrology, Landesklinikum Wiener Neustadt, Wiener Neustadt, Austria; 3 Department of Emergency Medicine, Medical University of Vienna, Vienna, Austria; 4 Department of Trauma Surgery, Medical University of Vienna, Vienna, Austria; 5 Department of Internal Medicine III, Medical University of Vienna, Vienna, Austria; 6 Department of Anesthesia, Intensive Care Medicine and Pain Medicine, Medical University of Vienna, Vienna, Austria; 7 Department of Neurology, Medical University of Vienna, Vienna, Austria; 8 Department of Clinical Pharmacology, Medical University of Vienna, Vienna, Austria; 9 Department of Internal Medicine I, Medical University of Vienna, Währinger Gürtel 18–20, 1090 Vienna, Austria

**Keywords:** NOACs, DOACs, Dabigatran, Factor Xa inhibitors, Consensus

## Abstract

The non-vitamin K antagonist oral anticoagulants (NOACs) have considerably changed clinical practice and are increasingly being used as an alternative to vitamin K antagonists (VKAs) for 3 main reasons: 1) an improved benefit-risk ratio (in particular lower rates of intracranial bleeding), 2) a more predictable effect without the need for routine monitoring, and 3) fewer food and drug interactions compared with VKAs. Currently, there are four NOACs available: the factor Xa inhibitors apixaban, edoxaban, and rivaroxaban, and the thrombin inhibitor dabigatran. This consensus paper reviews the properties and usage of NOACs in a number of high-risk patient populations, such as patients with chronic kidney disease, patients ≥80 years of age and others and provides guidance for the use of NOACs in patients at risk of bleeding.

## Introduction

Since the non-vitamin K antagonist oral anticoagulants (NOACs) became available, their use increased and they continued to replace vitamin K antagonists (VKAs), specifically in patients with atrial fibrillation (AF) and in those with venous thromboembolism (VTE). The NOACs show an improved benefit-risk ratio with less intracranial bleeding, a more predictable effect without the need for routine monitoring, and fewer food and drug interactions compared with VKAs [1]. Currently, there are 4 NOACs available: the factor Xa inhibitors apixaban, edoxaban and rivaroxaban and the thrombin inhibitor dabigatran. Table 1 gives an overview of pharmacokinetic and pharmacodynamic key properties of the currently available NOACs.

**Table 1:** Key properties of the available NOACs

	Dabigatran	Rivaroxaban	Apixaban	Edoxaban
*Mechanism of action*	Direct thrombin inhibition	Direct factor Xa inhibition	Direct factor Xa inhibition	Direct factor Xa inhibition
*Bioavailability*	6.5%	80–100%^a^	50%	62%
*Prodrug*	Yes	No	No	No
*Interaction with food intake*	No	Should be taken with a meal^b^	No	No
*Renal excretion of absorbed dose*	80%	35%	27%	50%
*Median elimination half-life in patients with normal renal function* ^*c,d*^	12–17 h	5–9 h (younger patients) 11–13 h (older patients)	12 h	10–14 h
*T*_*max*_	0.5–2 h	2–4 h	3–4 h	1–2 h
*Protein binding*	34–35%	92–95%	87%	55%
*Liver metabolism: CYPA4 involved*	No	Yes (hepatic elimination ~18%)	Yes (elimination 25%)	Minimal (elimination <4%)
*Absorption with H*_*2*_ * blockers/PPI*	Reduction of 12–30% (not clinically relevant)	No effect	No effect	No effect
*Asian ethnicity*	AUC (steady state) increased by 25%	No effect	No effect	No effect

*AUC* area under the curve, *PPI* proton pump inhibitors, *Tmax* time to reach maximal plasma concentration

^a^If taken with a meal

^b^For the 15 and 20 mg dosages

^c^According to [1]

^d^For changes of half-lives with decreasing renal function, see Table 4, references: [1–5] and labels

At the moment there are two main indications for the use of NOACs: treatment and secondary prevention of VTE, e.g., deep vein thrombosis and pulmonary embolism, and prophylaxis of thromboembolism in non-valvular AF (NVAF). In patients with valvular AF (e.g., moderate to high-grade mitral stenosis and patients with mechanical heart valves; [6]), NOACs are not recommended [7]. Due to the overall increasing use of NOACs and their favorable safety profile compared to VKAs, NOACs are frequently prescribed in patients with an increased risk of bleeding. The current consensus document will provide guidance for the use of NOACs in these patients. It should be noted that some of the recommendations given here might be off-label. In such situations, the patient must be informed and written informed consent should be obtained before the initiation of treatment.

## Chronic kidney disease (CKD)

### Recommendations

The recommended dosages of NOACs in CKD should be used (Tables 2, 3 and 4).

Calculation of the estimated glomerular filtration rate (eGFR) by the Cockroft-Gault formula.

Comment: in the large NOAC studies the Cockroft-Gault formula was used to estimate GFR. Therefore, dose adjustments of NOACs according to renal function should primarily be based on this formula; however, since the publication of the latest Kidney Disease—Improving Global Outcome (KDIGO) guidelines [8], another formula, the 2009 CKD-EPI (chronic kidney disease epidemiology collaboration) formula, is recommended for reporting eGFR. Physicians must be aware of the differences between the estimated GFR values obtained with these formulas and of the fact that the Cockroft-Gault formula overestimates kidney function, especially if GFR is below 60 ml/min.

The different renal excretion rates of the NOACs should be considered (Table 4*).*

Renal function should be monitored regularly. The monitoring intervals depend on the stability of renal function and on factors that might have a negative impact (e.g., intercurrent infections). As a general rule, the monitoring interval (in months) can be calculated by dividing the GFR (in ml/min) by 10; e.g.: if the GFR is 40 ml/min, 40/10 = 4, therefore the renal function should be monitored at least every 4 months, unless there were events potentially leading to an acute worsening of kidney function.

**Table 2:** Dosing in chronic kidney disease and nonvalvular atrial fibrillation (NVAF)

Creatinine clearance (CrCl)	Dabigatran	Rivaroxaban	Apixaban	Edoxaban
CrCl < 15 ml/min	No	No	No	No
CrCl 15–29 ml/min	No	1 × 15 mg^b^	2 × 2.5 mg^c^	1 × 30 mg^d^
CrCl 30–50 ml/min	2 × 150mg^a^	1 × 15 mg	2 × 5 mg	1 × 30 mg
CrCl > 50 ml/min	2 × 150mg^a^	1 × 20 mg	2 × 5 mg	1 × 60 mg

References: summary of product characteristics Pradaxa®, Xarelto®, Eliquis®, Lixiana®

^a^In patients with high bleeding risk, in patients ≥80 years and in those concomitantly taking verapamil 2 × 110 mg should be used

^b^Use with caution

^c^This dosage should also be used if serum creatinine is ≥1.5 mg/dl (133 µmol/l) and either one of the following criteria is fulfilled: age ≥80 years or weight ≤60 kg

^d^This dosage also applies to patients ≤60 kg and patients taking P‑glycoprotein inhibitors like cyclosporine, dronedarone, erythromycin and ketoconazole

**Table 3:** Dosing in chronic kidney disease and acute venous thromboembolism (VTE)

Creatinine clearance (CrCl)	Dabigatran^a^	Rivaroxaban	Apixaban	Edoxaban^a^
CrCl <15 ml/min	No	No	No	No
CrCl 15–29 ml/min	No	**2** **×** **15** **mg**^c,d^ 1 × 20 mg^e,f^	**2** **×** **10** **mg**^c,g^ 2 × 5 mg^c,h^	1 × 30 mg^i^
CrCl 30–50 ml/min	2 × 150 mg^b^	**2** **×** **15** **mg**^d^ 1 × 20 mg^e,f^	**2** **×** **10** **mg**^g^ 2 × 5 mg^h^	1 × 30 mg
CrCl >50 ml/min	2 × 150 mg^b^	**2** **×** **15** **mg**^d^ 1 × 20 mg^e,f^	**2** **×** **10** **mg**^g^ 2 × 5 mg^h^	1 × 60 mg

References: summary of product characteristics Pradaxa®, Xarelto®, Eliquis®, Lixiana®

The higher initial doses for rivaroxaban and apixaban are indicated in bold type

^a^Dabigatran and edoxaban can only be used in acute VTE after initial therapeutic anticoagulation with low molecular weight heparin (LMWH) for ≥5 days

^b^In patients with high bleeding risk, in patients ≥80 years and in those concomitantly taking verapamil 2 × 110 mg should be used

^c^Use with caution

^d^For the first 3 weeks

^e^After the first 3 weeks

^f^Reduce to 1 × 15 mg if the bleeding risk exceeds the risk of VTE

^g^For the first treatment week

^h^After the first treatment week

^i^This dosage also applies to patients ≤60 kg and patients taking P‑glycoprotein inhibitors like cyclosporine, dronedarone, erythromycin and ketoconazole

**Table 4:** Renal excretion rates and elimination half-lives of non-vitamin K antagonist oral anticoagulants in different chronic kidney disease stages

Substance	Dabigatran^a^	Rivaroxaban	Apixaban	Edoxaban
*Percentage of renal excretion in patients without CKD*	80%	35%	27%	50%
*Half-lives, depending on CrCl*				
*CrCl* *>* *80* *ml/min*	12–17 h	5–9 h (young) 11–13 h (elderly)	12 h	10–14 h
*CrCl 50–80* *ml/min*	~17 h	~8.7 h	~14.6 h	~8.6 h
*CrCl 30–49* *ml/min*	~19 h	~9 h	~17.6 h	~9.4 h
*CrCl 15–29* *ml/min*	~28 h	~9.5 h	~17.3 h	~16.9 h

Reference: modified from [9]

*CKD* chronic kidney disease, *CrCl* creatinine clearance

^a^Dabigatran is the only NOAC of these 4 that can be removed by dialysis

### Introduction

Patients with chronic kidney disease (CKD) are particularly vulnerable to bleeding or thrombotic complications. The influence of kidney function on the coagulation system depends on the CKD stage. In advanced stages there is an increased bleeding risk as well as an increased risk for thrombosis. With progressing CKD, the risk of thrombosis increases more than the bleeding risk; however, in hemodialysis patients the bleeding risk exceeds the thrombotic risk. Therefore, decision-making in dialysis patients with an indication for oral anticoagulation is particularly challenging [10–17].

The role of CKD as a risk factor for the development of bleeding complications is emphasized by the inclusion of CKD in various scoring systems for the assessment of the bleeding risk, e.g., the HAS-BLED score [18].

### Renal function und NOAC trials

Of note, there is a new classification of CKD, based on the eGFR (stages G1–G5) as well as the stage of albuminuria (A1–A3; [8]). Although patients with an eGFR <25 ml/min were excluded from the major NOAC trials, the labels of all NOACs except dabigatran provide dosages for patients with an eGFR between 15 and 29 ml/min. Similarly, the current AF guidelines of the European Society of Cardiology (ESC) state that there are no controlled trials of NOACs in patients with a creatinine clearance (CrCl <25–30 ml/min); however, they do not state clearly whether or not NOACs should be used in patients with an eGFR between 15 and 29 m/min [7]. An Austrian consensus paper argues against the use of NOACs in patients with an eGFR <30 m/min [2]. In our opinion, CKD stage and not eGFR should be used in recommendations and guidelines. For instance, in the RE-LY study, an arbitrary eGFR lower limit of 50 ml/min was used [13]; however, that is not a cut-off between different CKD stages. More importantly, eGFR represents a dynamic parameter depending on many constantly changing factors in patients’ state of health, thus it has to be monitored closely in patients on NOAC therapy. Furthermore, although eGFR depends on the formula used for calculation, in many studies it is not clear which formula was used. Yet, for the same serum creatinine value, eGFR differs significantly, based on the formula applied (see Table 5).

**Table 5:** Differences in eGFR values, according to formula used for a 67-year old, Caucasian woman with a body weight of 80 kg

Creatinine (mg/dl)	eGFR according to formula		
	Cockroft-Gault	MDRD	CKD-EPI
1.1	63	50	52
1.2	57	40	47
1.8	38	28	28.7

*GFR* glomerular filtration rate, *MDRD* modification of diet in renal disease, *CKD-EPI* chronic kidney disease epidemiology collaboration

As shown in Table 5, according to the Cockroft-Gault formula, more patients would qualify for NOAC treatment than according to the MDRD and CKD-EPI formulas. According to the KDIGO guidelines, the CKD-EPI formula is recommended to evaluate renal function [8]; however, as already mentioned the large randomized NOAC trials applied the Cockroft-Gault formula to assess CKD and therefore this formula should be used to estimate GFR to decide if the NOAC dosage needs to be reduced. It should be mentioned here that in the case of apixaban, dose adjustment is not based on GFR. A lower dosage of apixaban should be used if serum creatinine is ≥1.5 mg/dl (133 µmol/l) and either one of the following criteria is fulfilled: age ≥80 years or weight ≤60 kg. Important pharmacokinetic differences between the available NOACs must be taken into account (Table 1). Subanalyses of the large randomized NOAC trials which compared the efficacy and safety of the VKA warfarin with NOACs in patients with CKD and atrial fibrillation reported the following:

- Patients with CKD in the ARISTOTLE trial demonstrated an increased risk of bleeding and adverse cardiovascular events compared to patients with normal renal function [14]. Compared to warfarin, patients taking apixaban had lower rates of stroke, death and major bleeding, regardless of renal function. There was a significant reduction of major bleeding in the apixaban arm with an eGFR of <50 ml/min resulting in an absolute risk reduction of 3.2% (number needed to treat: 31).
- In the RE-LY study, the rates of stroke or systemic embolism, major bleeding and all-cause mortality increased as renal function decreased. The rates of stroke or systemic embolism were lower with dabigatran 150 mg and similar with 110 mg twice daily compared to warfarin, without significant heterogeneity in subgroups defined by renal function (interaction *p* > 0.1 for all). For the outcome of major bleeding, there were significant interactions between treatment and renal function according to CKD-EPI and MDRD equations (*p* < 0.05). The relative reduction in major bleeding with either of the tested dabigatran dosages compared to warfarin was greater in patients with GFR ≥80 ml/min when compared with warfarin [13].
- The relative efficacy of edoxaban in the prevention of arterial thromboembolism decreased with higher creatinine clearance rates.; however, the bleeding rates with edoxaban were lower than with warfarin in all examined ranges of creatinine clearance (30–50 ml/min, >50–95 ml/min, >95 ml/min; [15]).
- With rivaroxaban, the bleeding rates in AF patients were similar to those with warfarin in all examined CKD stages (CrCl 30–49 ml/min and CrCl >50 ml/min), but fatal bleeding occurred more often in the warfarin arm (0.74 vs. 0.28; *p* = 0.047; [17]). For rivaroxaban, there is a subanalysis of the EINSTEIN DVT and EINSTEIN PE trials for patients with renal impairment [16]. It shows that with decreasing renal function, the risk of recurrent VTE increases. In patients receiving enoxaparin/VKA, the risk of major bleeding increases with the decrease of renal function; however, this is not the case with rivaroxaban.
- It should be noted that there is no dose reduction for apixaban and rivaroxaban in CKD patients with DVT (Table 3).

In summary, compared to warfarin, the net clinical benefit for rivaroxaban and edoxaban remains roughly the same, regardless of renal function [15–17]. For apixaban, the benefit increases with decreasing renal function [14], whereas for dabigatran it decreases with decreasing renal function [13].

### Remaining questions

Although kidney function has been the subject of several studies and recommendations, there are currently no data on the use of NOACs in patients with massive proteinuria leading to nephrotic syndrome. It is to date unknown whether and how pharmacokinetics or pharmacodynamics (PK/PD) of NOACs are influenced by proteinuria. Currently, a phase 1/2 trial on PK/PD of apixaban is being conducted in patients with nephrotic syndrome [19]. The considerable differences in the rates of protein binding between the various NOACs could be of relevance in the context of nephrotic syndrome due to hypoproteinemia.

Another open question is how to deal with CKD patients of older age, relevant comorbidities or acute worsening of the health condition. For instance, there are data showing that 23% of patients hospitalized with acute heart failure experience a worsening of renal function which remains permanent in 10% and is transitory in 13% [20]. (A transitory worsening of renal function in this study was defined as an increase of serum creatinine by ≥0.3 mg/dl and/or by ≥25% from baseline at the index hospitalization which did not persist until the last measurement before discharge). Patients with unstable kidney function need close monitoring since there might be a need for dose adjustment or withdrawal of the NOAC. The frequency of evaluating renal function also depends on the clinical situation. In cases of acute clinical problems (e.g., intercurrent infections), renal function should be monitored more closely. Furthermore, interactions with other substances might significantly affect plasma levels of NOACs (e.g., some antibiotics and antimycotics). In such cases, therapeutic drug monitoring for the NOAC should be considered.

In patients with CKD stage 5 who are already on or about to be put on renal replacement therapy, there is a clear contraindication for NOACs; however, new pharmacological data indicate that apixaban [21] as well as rivaroxaban (15 mg/day; [4]), both showing the lowest renal excretion rates among NOACs [1], might be used without causing major problems in hemodialysis patients [4, 21]. Of note, patient numbers in these analyses were extremely low. Thus, the use of apixaban and rivaroxaban in the dialysis population cannot be recommended at this point.

## Old age (>80 years)

### Recommendations

There are specific recommendations for dose reduction of dabigatran and apixaban^1^ in patients ≥80 years, but not for rivaroxaban and edoxaban.

Patients ≥80 years who receive a NOAC should be monitored more closely.

The use of oral anticoagulation in this patient group must be decided on an individual basis. However, when assessing the net benefit for an individual patient, the higher risk of thromboembolic events should also be taken into account.

### Introduction

Biological age can vary considerably and does not necessarily match chronological age. Patients ≥80 years of age represent a special population for a number of reasons. First, there is a constant decline in renal function with increasing age (see Section “Chronic kidney disease”) and one should bear in mind that renal function is a very dynamic parameter. Furthermore, impairment of liver function is common in the elderly and can be monitored by synthesis parameters like the prothrombin time. Finally, an increased risk of falls and osteoporosis results in an elevated risk of bone fractures.

### NOAC dosages

Several NOAC labels include specific instructions for dose reductions for patients ≥80 years. For dabigatran, dosage should be reduced to 2 × 110 mg daily in VTE and NVAF, if patients are ≥80 years. In contrast, the dose adjustment of apixaban depends on the indications and further criteria. For NVAF, the apixaban dose should be reduced to 2 × 2.5 mg in patients ≥80 years, if 1 of the following 2 other criteria is met: body weight ≤60 kg or serum creatinine ≥1.5 mg/dl. For VTE, no dose reduction is recommended for apixaban. For rivaroxaban and edoxaban, no dose adjustment is recommended for patients ≥80 years.

### Appropriateness of anticoagulation

A recent study tried to determine whether the rates of oral anticoagulation in frail older adults with AF are appropriate or not and how useful risk prediction scores are in this context [22]. For 225 residents of a nursing home with a clinical frailty scale score ≥5, CHA_2_DS_2_-VASc [1] and HAS-BLED scores were retrospectively calculated. It turned out that bleeding risk was relatively low whereas stroke risk was high. Nevertheless, only 20% of the patients were orally anticoagulated.

In summary, in patients ≥80 years the decision for oral anticoagulation must be made on an individual basis. Several factors (biological age, frailty, dementia, comorbidities, renal and liver function, bleeding risk etc.) should be considered. Most importantly, if a patient ≥80 years is put on a NOAC, this patient should be monitored closely (renal and liver function, nutritional status, consider use of a frailty score, blood in feces, cognitive impairment).

## Patients with coagulation disorders

### Recommendations

In hereditary coagulation disorders anticoagulation increases the risk of bleeding and possible disadvantages and advantages of treatment with NOACs have to be assessed in every individual patient Whether anticoagulation therapy is possible in an individual patient depends on the degree of the coagulation disorder. In patients with severe forms of hemophilia, von Willebrand disease or other severe clotting disorders, anticoagulation is considered as contraindicated by many experts. In thrombocytopenia, therapeutic anticoagulation may be used above platelet counts of 50 G/l, whereas below a platelet count of 20 G/l, any anticoagulation is contraindicated. In patients with platelet counts between 20 and 50 G/l anticoagulation may be considered only in very specific situations on a case by case basis and preferably prophylactic doses of heparins should be used. If a patient presents with acute VTE and cannot be anticoagulated because of very high bleeding risk, a vena cava filter may be considered as an alternative. Likewise, occlusion of the left atrial appendage may be used in patients with AF and is a contraindication for anticoagulation.

### Introduction

Patients with hereditary or acquired disorders of the blood coagulation system, with thrombocytopenia or platelet function disorders are often at an increased risk of bleeding. The most prevalent disorders are mentioned here.

### Hemophilia

As the age of the hemophilia population rises, the prevalence of NVAF in hemophilic patients is likely to rise as well. In a report from 2014 the general prevalence of NVAF in people with hemophilia (PWH) in Europe was 0.8%, rising to 3.4% in patients older than 60 years [23]. A European consensus statement focused on the management of AF in PWH [24]. Stroke risk in PWH seems to be lower than in the general population; however, this may only be true for patients with severe hemophilia [25] as this effect was not found in non-severe hemophilia. Consequently, the lower stroke rate in PWH might increase in patients with factor replacement therapy [24].

There is agreement that NVAF in PWH should be treated by a multidisciplinary team, with a hematologist leading anticoagulation therapy [24]. The CHA_2_DS_2_Vasc score should be used, but it may overestimate stroke risk in PWH [24]. Conversely, the HAS-BLED score should not be used in PWH as it would substantially underestimate the bleeding risk [24]. Oral anticoagulation with VKAs or NOACs may be considered in PWH with a very high risk of stroke if trough levels of FVIII/IX are adequate (Table 6; [24]). The definition of very high risk has to be determined individually and cannot be determined by a single parameter [24]. Of note, there are no data for the use of NOACs in PWH.

**Table 6:** Suggested minimum trough levels of FVIII/IX considered safe for anticoagulation treatment in different settings

Setting	Mean value (IU/ml)	Range
Antiplatelet monotherapy	0.035	0.01–0.1
VKAs	0.24	0.1–0.5
Dual antiplatelet therapy	0.14	0.04–0.3
NOACs	0.23	0.1–0.5
Cardioversion with concomitant therapeutic doses of heparin	0.40	0.1–0.8
During transesophageal echocardiography	0.30	0.01–0.8

Reference: [24]

*VKAs* vitamin K antagonists, *NOACs* non-vitamin K antagonist oral anticoagulants

### Von Willebrand disease

Von Willebrand disease (vWD) is one of the most frequent clotting disorders; however, it is still rare with approximately 0.01% clinically affected cases in the population [26]. Von Willebrand factor (vWF) levels have been shown to be increased in patients with NVAF [27], thus is can be assumed that also in patients with a low vWF the levels increase, not only with age but also when they have NVAF. There is a great variability of severity, with severe forms being very rare, and the risk of stroke in patients with vWD is unknown. The cut-offs for anticoagulation suggested by experts in patients with hemophilia might also be applicable for the vWF levels (Table 6); however, as in other bleeding disorders, a case by case decision, balancing the risk of bleeding with the risk of thrombosis or stroke, has to be made for each individual patient with vWD.

### Thrombocytopenia

Data on thrombocytopenia and anticoagulation are scarce [28]. In guidelines for treatment of patients with cancer, a platelet count of >50 G/l is considered as appropriate for therapeutic anticoagulation [29]. This cut-off has also been used in recent studies [30]. In patients with platelet counts between 20 and 50 G/l anticoagulation may be considered only in very specific situations on a case by case basis and preferably prophylactic doses of heparins should be used. Below a platelet count of 20 G/l, anticoagulation is contraindicated.

In patients with autoimmune thrombocytopenia, if there is a strong indication for anticoagulation, the risk of a thrombotic event should be evaluated (e.g., CHA_2_DS_2_-VASc score, if applicable). If there is an indication for temporary anticoagulation (as in some VTE cases), a short-term anticoagulation (e.g., 3 months) should be used; however, if long-term anticoagulation is needed in patients with autoimmune thrombocytopenia, treatment options that are capable of increasing the platelet count should be considered [31]. If a patient with high bleeding risk presents with acute VTE, a vena cava inferior filter may be considered as an alternative to anticoagulation [32].

## Patients with acute bleeding

### Recommendations

In patients with acute major or clinically relevant non-major bleeding, any kind of anticoagulation therapy is strictly contraindicated.

An antagonist for dabigatran, idarucizumab, is available and recommended. An antagonist for factor Xa inhibitors, andexanet alpha, has so far only been approved for clinical use in patients in the USA. Therefore, prothrombin complex concentrates are still recommended for the reversal of factor Xa inhibitors in cases of life-threatening bleeding in the Europe.

In patients with acute severe bleeding, no anticoagulation of any kind should be used. A recent position paper of the European Society of Cardiology (ESC) includes an algorithm on how to deal with the reversal of NOACs (e.g., bleeding, emergency surgery; [33]). First of all, there has to be a decision whether or not a reversal of the NOAC is necessary. In any case, administration of the NOAC should be stopped and general measures (e.g., mechanical compression, hemodynamic support, volume replacement etc.) taken. If deemed necessary, specific reversal agents should be used. In the case of dabigatran, hemodialysis could be considered.

### Antagonization of NOACs

European guidelines on the management of major bleeding and coagulopathy following trauma recommend that in acute bleeding of patients on NOACs the plasma level of the NOAC should be measured [34]. A position paper about reversal strategies for NOACs by the ESC Working Group on Cardiovascular Pharmacotherapy and the ESC Working Group on Thrombosis is also available [33].

For dabigatran, there is an antidote available. The Fab antibody fragment idarucizumab binds to dabigatran in a 1:1 ratio and is licensed as a specific antidote to dabigatran [35]. Usually, a single dose of 5 g idarucizumab, which is rapidly excreted by the kidney is sufficient as an antagonist to dabigatran. Dabigatran plasma concentrations could increase again within 24 h, possibly due to a shift of dabigatran from the extracellular space to the circulation, demanding a second administration of idarucizumab [36]. In the REVERSE-AD study 1.8% of patients received a second dose of the antibody [37]. Readministration of dabigatran 24 h after administration of idarucizumab is possible. An impairment of renal function does not seem to influence the effect of idarucizumab on dabigatran, although idarucizumab exposure (as well as dabigatran concentration) increases with decreasing renal function. Particularly, older patients and patients with reduced renal function need the full dose of 5 g idarucizumab [38]. After the administration of idarucizumab, dabigatran plasma levels should be checked again in order to measure the efficacy in the individual patient, particularly if there is insufficient clinical hemostasis [39, 40].

The specific antidote for factor Xa inhibitors, i.e. andexanet alpha, has so far only been approved for clinical use in patients in the USA. Until andexanet alpha becomes available in Europe, high-dose prothrombin complex concentrates (PCCs; dose of 50 U/kg) should be used to antagonize factor Xa inhibitors if necessary [34]. In the latter case, one should be aware of an increased thromboembolic risk. If the patient is bleeding, intravenous tranexamic acid (15 mg/kg or 1 g) should additionally be considered.

In emergency situations, specific tests should be used. For dabigatran, the Hemoclot™ test (Hyphen Biomed, Neuville-sur-Oise, France) or the ecarin clotting time should be used; for factor Xa inhibitors, calibrated anti-Xa activity tests are recommended. If those are not available, for dabigatran (diluted) thrombin time, and for factor Xa inhibitors non-calibrated anti-Xa tests may be used to exclude residual thrombin inhibition and anti-Xa activity, respectively; however, these tests are not sufficient to guide therapy.

In patients who survived the acute situation a comprehensive evaluation of a restart of anticoagulation should be performed. Particularly in patients with a reversible bleeding risk factor, anticoagulation should be restarted as the risk of thromboembolism is still high in this vulnerable patient group. In patients with NVAF and an irreversible risk factor and therefore a contraindication for anticoagulation, an occlusion of the left atrial appendage should be considered [7].

## Patients with malignancies

### Recommendations

In cancer patients with VTE, low molecular weight heparins (LMWH) are safe and effective; however, edoxaban and rivaroxaban were non-inferior and superior to dalteparin with respect to recurrent VTE, respectively, whereas bleeding was found to be increased with both NOACs. After an initial treatment period of 3–6 months with LMWH, if anticoagulation is still indicated either VKAs or NOACs could be used to continue, if the active phase of the malignancy is over and the tumor is in a stable condition. In cancer patients with AF, there is a clear indication for oral anticoagulation with VKAs or (preferably) with NOACs.

Patients with malignancies have an increased risk of VTE (first time as well as recurrent events) as well as an increased risk of bleeding [41–44].

### Venous thromboembolism

In VTE patients with malignancies, LMWH was the gold standard of anticoagulation for a long time. It was shown that in cancer patients LMWH is more effective than VKA in preventing VTE recurrences without increasing the risk of bleeding [45]. A network meta-analysis suggested that NOACs are at least equivalent if not better than VKAs in cancer patients with VTE. The indirect network comparison between NOACs and LMWH indicated comparable efficacy and a non-significant relative risk reduction towards improved safety with NOACs. These results prevailed after adjusting for different risk of recurrent VTE and major bleeding between LMWH vs. VKA and NOAC vs. VKA studies [44].

A recent large randomized study (HOKUSAI VTE Cancer) demonstrated non-inferiority of a combined primary outcome of recurrent VTE and severe bleeding for edoxaban versus dalteparin [46]. Interestingly, on edoxaban recurrent events were less (not significantly), but there was an increased risk of gastrointestinal bleeding, primarily in patients with gastrointestinal tumors. Another randomized study (SELECT-D) with less study participants comparing rivaroxaban to dalteparin in patients with cancer was recently published [47]. In this study the recurrence rate of VTE was lower, but the risk of major and clinically relevant non-major bleeding was higher in patients receiving rivaroxaban. There were no significant differences in mortality in both studies [46, 47].

The available published international guidelines still emphasize that NOACs are not the first choice for VTE treatment or prophylaxis in cancer patients [48]; however, these guidelines were published before the HOKUSAI VTE Cancer study was available. The authors of this consensus agree that NOACs (especially edoxaban and rivaroxaban) are a potential alternative to LMWH. In particular, the increased bleeding rate in cases of gastrointestinal tumors has to be kept in mind, and in these patients the potentially higher bleeding risk has to be balanced against the higher convenience of an oral versus a subcutaneous drug. Moreover, in patients with thrombocytopenia caution with full dose NOACs is recommended. After an initial treatment period of 3–6 months with LMWH, if further anticoagulation is indicated, patients can be switched to VKAs or NOACs on the basis of individual choices, if the active phase of the malignancy is over and the tumor is in a stable condition [48].

Further studies directly comparing NOACs with LMWH ± VKA in secondary prophylaxis are under way [49–51].

### Atrial fibrillation

In patients with cancer and NVAF either NOACs or VKAs may be used for stroke prophylaxis. If NOACs are given, interactions with various cancer treatments should be taken into account [2].

## Surgery patients

### Recommendations

For patients on a NOAC who are not bleeding but need a surgical intervention, it is important to determine for how long this intervention can be delayed. If it is possible to wait until the NOAC is mostly eliminated (depending on renal function, specific substance and bleeding risk), this should be done. If it is not possible to wait, NOAC plasma levels should be estimated on the basis of calibrated assays and the NOAC should be antagonized (idarucizumab for dabigatran, prothrombin complex concentrates for factor Xa inhibitors). If it is not possible to wait for the NOAC plasma level, antagonization should be performed immediately.

This section deals with patients on NOACs who are not bleeding but need a surgical intervention (for surgical patients who are actively bleeding, see Section “Antagonization of NOACs”).

The subject of perioperative bridging is not discussed in this paper, as bridging is generally not necessary in patients receiving NOACs. In cases of required surgery during NOAC therapy, it needs to be evaluated whether surgery can be postponed or not. If yes, the suggested time interval between the last NOAC intake and surgery depends on the NOAC used and renal function (Table 7).

**Table 7:** Last intake of drug before elective surgical intervention

	Dabigatran		Apixaban/edoxaban/ rivaroxaban	
	Low risk (h)	High risk (h)	Low risk (h)	High risk (h)
CrCl > 80 ml/min	≥24	≥48	≥24	≥48
CrCl 50–80 ml/min	≥36	≥72	≥24	≥48
CrCl 30–49 ml/min	≥48	≥96	≥24	≥48
CrCl 15–29 ml/min	Not indicated	Not indicated	≥36	≥48

Reference: modified from [1]

*CrCl* creatinine clearance

If surgery cannot be postponed, the next step is evaluation of NOAC plasma levels. If there is no relevant plasma level (e.g., because the patient did not recently take the NOAC), surgery is immediately possible. If there are relevant plasma levels, the NOAC should be antagonized preoperatively (see Section “Antagonization of NOACs”).

If the surgical intervention must be performed immediately, the NOAC should be antagonized (see Section “Antagonization of NOACs”); however, even in this case an evaluation of NOAC levels should be initiated preoperatively, since this information which might presumably arrive during the intervention could be helpful to anesthesiologists and surgeons.

## Polypharmacy and interactions

### Recommendations

Human immunodeficiency virus (HIV) protease inhibitors strongly increase plasma concentrations of NOACs and this combination should therefore be avoided. The same applies to most azole antimycotics. Dabigatran should not be combined with the antiarrhythmic dronedarone or with immunosuppressives like cyclosporine or tacrolimus (AUC of the NOAC increases). Some anticonvulsives, like carbamazepine, phenobarbital and phenytoin, as well as St. John’s wort and rifampicin, strongly decrease plasma levels of NOACs and should therefore be avoided. Substances which pharmacodynamically interact with NOACs include other kinds of anticoagulants, antiplatelet drugs, glucocorticoids and selective serotonin reuptake inhibitors (SSRI).

A dose reduction can be necessary:

- for edoxaban, if co-administered with the P‑glycoprotein inhibitors cyclosporine, dronedarone, erythromycin or ketoconazole
- for dabigatran, if co-administered with verapamil

As so far there are only a few recommendations for routine measurement of NOAC plasma levels [52, 53], the potential for drug-drug interactions (DDI) is especially important to consider. There are 2 kinds of possible DDI: pharmacokinetic and pharmacodynamic interactions. A comprehensive overview of pharmacokinetic interactions is given in the new European Heart Rhythm Association guidelines [1]. Furthermore, there are pharmacodynamic interactions, e.g., the increase of bleeding time with NOAC + naproxen [1]. Other agents which pharmacodynamically interact with NOACs include other kinds of anticoagulants, antiplatelet drugs, glucocorticoids and SSRI.

## Stroke patients

### Recommendations

After a transitory ischemic attack (TIA) if an ischemic lesion and bleeding is excluded, NOAC therapy can be initiated or resumed immediately.

After a stroke, if bleeding is excluded and the NIH stroke score is ≤16, anticoagulation can be initiated or resumed after 4 days. After a stroke, if bleeding is excluded and the NIH stroke score is >16, anticoagulation can be initiated or resumed after 2 weeks. If there is an intracranial hemorrhage on NOAC therapy, the decision whether or not to resume oral anticoagulation is difficult, depends on many factors and has to be made individually.

One of the main questions concerning stroke and NOACs is how long does the interval between stroke and restart of NOAC therapy have to be? This question is difficult to answer from evidence because patients with a recent stroke or even TIA were excluded from almost all NOAC trials. The retrospective RAF study showed that for patients with AF and cardioembolic stroke the best time to initiate anticoagulation is between 4 and 14 days after the event [54]. This is considerably earlier than the 4–8 weeks recommended in current guidelines [7]; however, after a TIA, if there is no bleeding and no ischemic lesion in CT, anticoagulation may be started immediately. After a stroke, if bleeding is excluded and the score of the National Institutes of Health stroke scale (NIHSS) does not exceed 16, anticoagulation can be initiated on day 4. In more severe strokes (NIHSS > 16), anticoagulation can be initiated after at least 2 weeks.

**Fig. 1:**
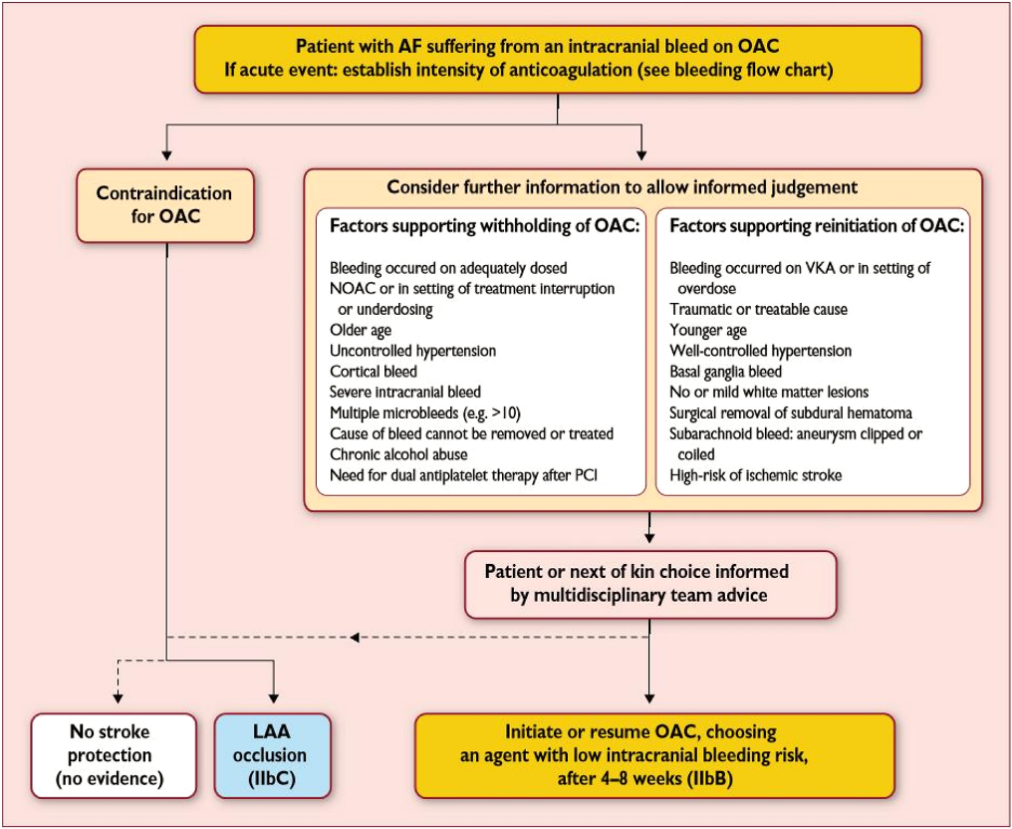
Initiation or resumption of anticoagulation in atrial fibrillation patients after an intracranial bleeding. *AF* atrial fibrillation, *LAA* left atrial appendage, *NOAC* non-vitamin K antagonist oral anticoagulant, *OAC* oral anticoagulation, *PCI* percutaneous coronary intervention, *VKA* vitamin K antagonist. (Reproduced with permission from the European Society of Cardiology [7])

In patients who develop intracranial hemorrhage during oral anticoagulation with a NOAC, anticoagulation has to be stopped immediately. Suggestions for the management of bleeding were stated in Section “Patients with acute bleeding”. Many factors have to be taken into account in order to decide whether or not oral anticoagulation can be resumed after 4–8 weeks. The ESC guidelines for the management of AF provide recommendations on how to proceed in these patients (Fig. 1).

However, if intracranial bleeding on OACs occurs spontaneously, i.e. there are no further precipitating factors, like hypertension or trauma, there is a clear contraindication to resume OAC after the bleeding event. In this case, occlusion of the left atrial appendage is a treatment option (requiring antiplatelet therapy). For further arguments to withhold OAC see Fig. 1.

## Antiplatelet therapy in patients with coronary heart disease (CHD) on NOACs

### Recommendations

The duration of dual or triple therapy (NOAC plus 1 or 2 antiplatelet agents) depends on stroke risk, bleeding risk and clinical presentation (stable coronary heart disease, CHD vs. acute coronary syndrome, ACS) as well as on the complexity of the coronary intervention. No more antiplatelet therapy should be given 1 year after an acute coronary event, and the NOAC should be continued as monotherapy. If triple therapy is initiated, the preferred combination at present is NOAC + aspirin + clopidogrel.

Decisions on dual and triple therapy should be made at the specialized center where the coronary event was treated [7]. In triple therapy, it is reasonable to prescribe the reduced dose of the respective NOAC; however, the standard dose should be resumed after terminating dual antiplatelet therapy. The reason for excluding prasugrel and ticagrelor from triple therapy is the higher bleeding risk these compounds have shown compared to clopidogrel in the TRITON TIMI-38 [55] and PLATO [56] trials.

The duration of triple therapy should be kept as short as possible according to the current guidelines considering the ischemic and bleeding risk of the individual patient. Recently, PIONEER AF PCI [57], the first randomized trial on triple or dual therapy with a NOAC compared to standard triple therapy with a VKA in NVAF, was published. The study showed that dual therapy with low-dose rivaroxaban (15 mg/day) + clopidogrel or triple therapy with very low dose rivaroxaban (2 × 2.5 mg/day) + aspirin + clopidogrel significantly reduced major bleeding compared to standard triple therapy with warfarin + aspirin + clopidogrel. Furthermore, the rate of ischemic events was comparable in patients with standard triple therapy and in those with both rivaroxaban regimens; however, it has to be kept in mind that the study was not adequately powered to confirm a non-inferiority of the two rivaroxaban regimens and triple therapy with a VKA regarding the prevention of thromboembolic events.

In the RE-DUAL PCI study, 2725 patients with NVAF who had undergone PCI were randomized to 1 of 2 treatment approaches: standard triple therapy, consisting of the VKA warfarin, a P2Y_12_ inhibitor (clopidogrel or ticagrelor) and aspirin (for 1–3 months) or dual therapy with dabigatran (110 mg or 150 mg, twice daily) and a P2Y_12_ inhibitor (clopidogrel or ticagrelor; [58]).

The primary endpoint was a composite of major and clinically relevant non-major bleeding during follow-up and occurred less frequently in both dabigatran treatment groups compared to the triple therapy group. Furthermore, the rate of ischemic events was comparable in patients with standard triple therapy and in those with dabigatran plus clopidogrel or ticagrelor; however, like the PIONEER AF PCI study, the RE-DUAL PCI study was not adequately powered to confirm a non-inferiority of the two dabigatran regimens (if evaluated separately) and triple therapy with a VKA regarding the prevention of ischemic events.

According to the recent guidelines of the ESC [59], patients undergoing peripheral endovascular interventions with stent implantation should receive dual antiplatelet therapy with aspirin and clopidogrel for at least 1 month, and long-term therapy with 1 antiplatelet agent. If these patients also have an indication for OAC, the treatment strategy should be chosen based on the individual bleeding risk. In cases of a high bleeding risk, the patient should be directly treated with a NOAC or VKA monotherapy after peripheral angioplasty and stenting while in the case of a low bleeding risk, a combination therapy consisting of OAC + aspirin or clopidogrel for at least 1 month and up to 12 months may be considered. After 12 months all patients, who underwent peripheral angioplasty with stenting and have an indication for OAC (NVAF or VTE) should receive VKA or NOAC monotherapy.

## Conclusion

The NOACs have become the preferred treatment option in most patients with VTE and NVAF due to their superior safety profile compared to VKAs; however, the correct administration of dose reduction criteria and contraindications for NOACs is of utmost importance to achieve the optimal benefit-risk ratio and outcomes, particularly in patients at an increased risk of bleeding.

## Footnotes

1 For apixaban, 1 of 2 other criteria must be fulfilled: serum creatinine ≥1.5 mg/dl or body weight ≤60 kg.
